# In Vivo and In Vitro Investigation of a Novel Gelatin/Sodium Polyacrylate Composite Hemostatic Sponge for Topical Bleeding

**DOI:** 10.3390/jfb14050265

**Published:** 2023-05-10

**Authors:** Nusrat Jahan, Md Sowaib Ibne Mahbub, Byong-Taek Lee, Sang Ho Bae

**Affiliations:** 1 Department of Regenerative Medicine, College of Medicine, Soonchunhyang University, Cheonan 31151, Republic of Korea; 2 Institute of Tissue Regeneration, Soonchunhyang University, Cheonan 31151, Republic of Korea; 3 Department of Surgery, Soonchunhyang University Cheonan Hospital, Cheonan 31151, Republic of Korea

**Keywords:** hemostatic agent, gelatin/sodium polyacrylate (GSp) scaffold, superabsorbent polymer, thrombin, hemocompatibility, topical bleeding

## Abstract

Designing a functional and efficient blood-clotting agent is a major challenge. In this research, hemostatic scaffolds (GSp) were prepared from the superabsorbent, inter-crosslinked polymer sodium polyacrylate (Sp) bound to a natural protein gelatin (G) loaded with thrombin (Th) by a cost-effective freeze-drying method. Five compositions were grafted (GSp0.0, Gsp0.1, GSp0.2, GSp0.3, GSp0.3-Th) where the concentration of Sp varied but the ratios of G remained the same. The fundamental physical characteristics that increased the amounts of Sp with G gave synergistic effects after interacting with thrombin. Due to the presence of superabsorbent polymer (SAP) swelling capacities in GSp0.3 and GSp0.3-Th surge forward 6265% and 6948%, respectively. Pore sizes became uniform and larger (ranging ≤ 300 μm) and well-interconnected. The water-contact angle declined in GSp0.3 and GSp0.3-Th to 75.73 ± 1.097 and 75.33 ± 0.8342 degrees, respectively, thus increasing hydrophilicity. The pH difference was found to be insignificant as well. In addition, an evaluation of the scaffold in in vitro biocompatibility with the L929 cell line showed cell viability >80%, so the samples were nontoxic and produced a favorable environment for cell proliferation. The composite GSp0.3-Th revealed the lowest HR (%) (2.601%), and the in vivo blood-clotting time (s) and blood loss (gm) supported hemostasis. Overall, the results showed that a novel GSp0.3-Th scaffold can be a potential candidate as a hemostatic agent.

## 1. Introduction

Hemostasis can be achieved by converting blood into stable and insoluble fibrin using the body’s natural coagulation mechanism, which is divided into primary hemostasis and the coagulation cascade. Basically, wound or tissue healing involves four major steps: hemostasis, inflammation, multiplication and remodeling [1]. Hemostasis is the incipient stage of healing at the injured site [2]. Conventional hemostats have limited effectiveness for acute traumas and burns. The most popular hemostatic medications are absorbable gelatin (G) sponges and microfibrillar collagen, which are frequently coupled with bovine thrombin (Th) [3]. To prevent rapid blood loss, formulated hemostatic agents can play a key role [4,5,6], but the fabrication of easy-to-use, inexpensive, safe and effective hemostatic agents are the main focus of this study.

Superabsorbent polymers (SAPs) have received a lot of attention due to their numerous applications in biology, biomedical tissue engineering to stimulate tissues, wound dressing [7] and in medical and pharmaceutical uses to remove body fluids [8,9]. An SAP is a three-dimensional crosslinked, linear, or branching polymer having a significant number of hydrophilic groups that can absorb physiological fluids, saline solutions, or water up to thousand times its own weight. A polymer consisting of two distinct monomers is known as a copolymer. Acrylic acid (AA) and its sodium salt (sodium acrylate; SA) together produce sodium polyacrylate (Sp) [10]. The interesting fact about the Sp molecule is that it creates a link between the two chains when the hydrogen on a carboxyl group (–COOH) on one chain reacts with a double bond (–C=C–) on an adjacent chain. Consequently, crosslinking occurs at numerous locations inside the polymer and makes a mesh-like structure. The ability of an adjacent polymer chain to crosslink with one another distinguishes this polymer from the majority of other types. The chain’s interlocking criteria keeps it from melting or breaking apart in water, which is a major reason for its high water absorbency [11,12]. Another reason is that a crosslinked Sp has less mechanical strength. The main mechanism of the superabsorbent water-holding polymer is osmosis. When it interacts with water, sodium ions tend to disperse evenly across the polymeric network and are replaced by water molecules. To maintain a balanced Na⁺ concentration between the polymer and the water, water enlarges the polymer network and swelling occurs [13]. Osmotic pressure and molecular forces help keep the water in place and the polymer continuously expands during swelling until it finds equilibrium and stops absorbing water [14]. Other applications for Sp include high mechanical and thermal stabilizers [15,16], hygiene products [17], excellent thickeners [18], environmentally friendly coatings and adhesives [19]. Similar to every other synthetic substance, an SAP is also frequently analyzed for toxicity and safety, as well as its impact on the environment. Conventional SAPs are neutral and inert. Scientifically, these materials cannot revert to their initial monomers, so they are not harmful. In this case, polymerization chemically transforms the initial harmful monomers into a non-toxic product [15,18,20,21,22]. The Food and Drug Administration (FDA) allows Sp as an oral food additive [23].

G, a natural polymer derived from collagen, is a widely distributed protein found particularly in cattle or pig skin, bone and connective tissue after denaturation and partial hydrolysis. It serves an essential function in hemostasis to aid the healing of wounds, and it directly support fibroblasts proliferation, adhesion and differentiation as well as the migration of keratinocytes [24]. Recent studies showed that G can trigger macrophages and has a significant blood-clotting capacity [25,26]. In general, G is considered to be a potent hydrogel for its ready availability, excellent cell-addition quality, substantial hydrophilicity, biocompatibility and biodegradability. [27,28,29,30]. G contains one positive and one negative charge in its structure, which improves its ability to form ionic interactions with Sp. No additional crosslinker was added. Here, this inter-crosslinked biomaterial was directly mixed with G. 

Numerous internal and external hemostatic compounds have FDA approval. Th is the internal, and G and Sp are the external ones [31]. Th is also an indispensable blood-clotting factor that matures fibrin complex and activates platelets that inhibit onsite bleeding [32]. It is believed that by incorporating active hemostatic Th with passive hemostatic materials, the scaffold’s hemostatic potential will be greatly enhanced.

According to recent investigations, an ultrafast self-gelling and wet adhesive hemostatic powder has been developed using polyethyleneimine/poly(acrylic acid)/Qatarized chitosan (PEI/PAA/QCS) [33]. However, PEI/PAA powder has demonstrated a low capacity to absorb water and requires additional modification for hemostatic application. Another study reported a novel solid/solution interface complexation method to afford a PAA/Polyvinylpyrrolidone (PVP) complex with high bio-adhesion [34]. The hydrogel gradually dissolves and decomposes in the body, but the hazards of viral infection or allergic reaction are not entirely removed. In addition, PAA on a thermoplastic elastomer (a polystyrene—polybutadiene—polystyrene triblock copolymer) via free-radical polymerization creates a strong and instantly swelling hydrogel for bleeding control [35]. AA is a toxic monomer so it should be emphasized that the residual monomers might result in potential biological harm. 

Herein, novel GSp sponges loaded with Th are designed to improve hemostatic activity. Scaffolds were built using cost-effective freeze-dried technology, a very reliable way to create biomaterials [36]. The composite is innovative because it has a superabsorbent feature that can accelerate swelling and boost onsite blood absorption while G significantly affects the development and proliferation of fibroblasts. Most significantly, the system is safe, easily replicable and accessible. To the best of our knowledge, Sp has not yet been thoroughly explored as a hemostatic agent. Thus, the GSp scaffold is a promising biomaterial to aid instant onsite bleeding treatments based on its qualities of rapid hemostasis, ease of use, low price, high hydrophilicity, exceptional cytocompatibility and good hemocompatiblity.

## 2. Materials and Methods

### 2.1. Materials

Gelatin from porcine skin powder gel strength 300g Bloom, Type A (Sigma-Aldrich, Co., St. Louis, MO, USA), crosslinked sodium polyacrylate (Sp) powder (Sigma-Aldrich, Chemie GmbH, St. Louis, MO, USA), Thrombin from bovine plasma lyophilized powder 1000U/mg (Sigma-Aldrich, St. Louis, MO, USA), bovine serum albumin (BSA; Sigma-Aldrich, St. Louis, MO, USA), di-methyl sulfoxide 99.5% (DMSO; Daejung chemicals Co., Gyeonggi-do, Siheung-si, Republic of Korea), green fluorescein isothiocyanate conjugated phalloidin (FITC; Sigma-Aldrich company, St. Louis, MO, USA), fetal bovine serum (FBS; Thermo Fisher Scientific, Waltham, MA, USA), Dulbecco’s modified medium (Gibco), penicillin as well as streptomycin PS: 100U/mL (Thermo Fisher Scientific, Waltham, MA, USA), Gibco^TM^ trypsin-EDTA (Thermo-Fischer Scientific. Manassas, Waltham, MA, USA), Gibco^TM^ 3-[4.5-dimethylthiazol-2-yl]-2,5-diphenyltetrazolium bromide (MTT; Thermo Fisher Scientific. Anaheim, Waltham, MA, USA), 99.5% ethyl alcohol (Daejung chem. Co., Siheung-si, Republic of Korea), phosphate buffer saline tablets (PBS: Sigma-Aldrich, St. Louis, MO, USA), 4% para-formaldehyde (PFA; Sigma-Aldrich, St. Louis, MO, USA), LIVE/DEAD™ viability/cytotoxicity kit for mammalian cells (Thermo Fisher Scientific, Waltham, MA, USA), mouse L929 fibroblast cells (Korea cell line bank). Ten-week-old male Sprague–Dawley (SD) rats were used for the in vivo study (Dayoon Animal Experiment Center, Korea). 

### 2.2. Fabrication of Novel Gelatin/Sodium Polyacrylate (GSp) Scaffolds

**Scheme 1:**
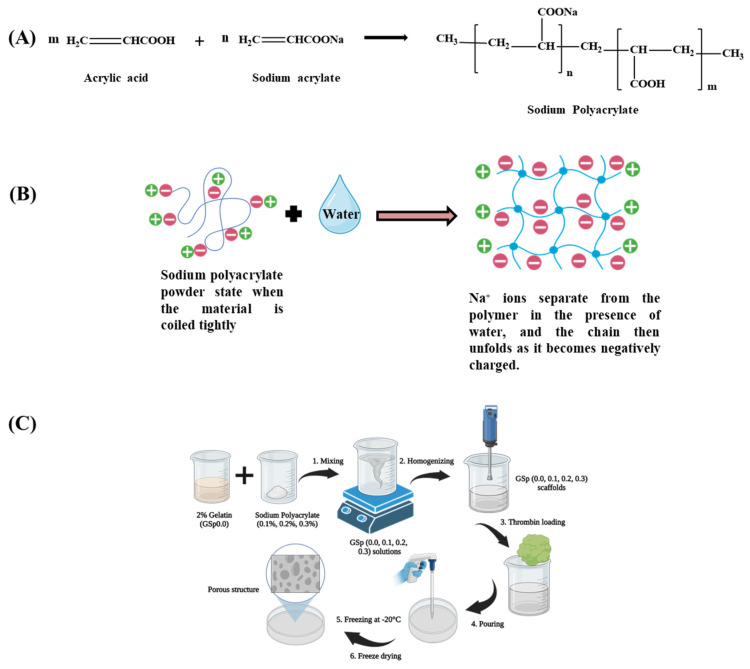
GSp0.2, GSp0.3, GSp0.3-Th scaffolds using Biorender. (**A**) Sodium polyacrylate’s internal crosslinked polymer structure, (**B**) Molecular state of Sp powder before and after reacting with water, (**C**) Schematic illustration of fabrication of Gelatin/Sodium polyacrylate (Gsp0.0, GSp0.1, GSp0.2, GSp0.3, GSp0.3-Th) scaffolds using Biorender.

To prepare freeze-dried GSp hemostatic sponge Type A, 2 g G was dissolved in 100 mL sterilized DI water and heated to 60 °C while stirring continuously for 30 min. Here, an inter-crosslinked Sp polymer was directly used. Three different concentrations of Sp (0.1, 0.2, 0.3 g) were weighted and vigorously mixed with G solution using a magnetic stirrer for 2 h as shown in Scheme 1C. A highspeed homogenizer (LK Lab, HG1010) was run for 5 min to ensure complete homogenization. Then Th powder was dissolved in distilled water. Th loaded in GSp0.3 at 50 NIH/mL concentration. Different compositions are labeled in Table 1. Equal volumes of GSp0.0, GSp0.1, GSp0.2, GSp0.3, GSp0.3-Th slurry (10 mL) was poured into (35 × 10 mm) crystal-grade polystyrene petri dishes. The samples were then frozen at −20 °C for 12 h and −80 °C for 24 h to avoid phase separation. Finally, they were freeze-dried for 48 h. 

The labeling and contents of the GSp scaffolds are presented in Table 1. The amount of G stayed the same while Sp quantity rose. Th was loaded only with GSp0.3.

### 2.3. Physical Characterization

#### 2.3.1. Microstructure Detection, EDS and Pore Diameter Inspection

After preparing the freeze-dried GSp sponges, cross-sections were made using a microtome blade. The newly produced lyophilized microstructures were examined via scanning electron microscopy (SEM, JEOL, JSM-6701F, 197 Tokyo, Japan). At first, samples were placed into the sample stage of SEM and coated with platinum sputter coater (Cressington Scientific Instrument, Watford, UK). An accelerated voltage of 10 kV was applied to understand the morphology of scaffolds through SEM images. EDS was performed to identify the elements inside the samples. Pore-size distribution was analyzed using ImageJ Fiji software (ImageJ 1.53t, National Institutes of Health, NIH, Bethesda, MD, USA). 

#### 2.3.2. Fourier Transform Infrared Spectroscopy (FTIR)

The spectrum of the composites and raw materials were identified by Fourier Transform Infrared Spectroscopy (Nicolet Ia10, Thermo-Fischer Scientific). The samples were examined over a wavelength range of 600–4000 cm^−1^ using OMNIC 7.3 spectra software at a resolution of about 8 cm^−1^.

#### 2.3.3. Swelling Rate (%) of Scaffolds

PBS uptake behavior was determined by the conventional weighing method. Briefly, all dry scaffolds (GSp0.0, GSp0.1, GSp0.2, GSp0.3, GSp0.3-Th) were weighted and cut into 1× 1 cm squares, representing (W_dry_) then placed in separate petri dishes after immersion in 5 mL PBS (W_wet_). Next, the samples were kept in an incubator at 37 °C. After 1, 5, 10 and 30 min the wet weights of the scaffolds were remeasured. The Swelling rate (%) was calculated by Equation (1):

(1) $$\Delta \;W\%=\frac{\mathrm{Wwet}\;-\;\mathrm{Wdry}}{\;\mathrm{Wdry}}\times 100\;$$

#### 2.3.4. pH Sensitivity Evaluation

The difference in pH alteration was assessed by soaking the grafts in 5 mL PBS. The Initial pH of PBS was 7.4 when interacting with the sponges. After 1, 3, 5 and 7 days, it was measured using a pH meter (Orion star A211, Thermo-Fisher Scientific). No additional PBS was added during the immersion process.

#### 2.3.5. Contact Angle Measurement

Hydrophilicity or hydrophobicity was evaluated using the wettability measurement apparatus that exploits sessile drop method (Drop-shape analyzer, DSA 100, KRUSS, Hamburg, Germany). A stainless-steel needle was used to drop 5 μL of distilled water over the scaffold surface, which was firmly attached to a flat base. A CCD video camera captured angular deflections every 20 s. Afterwards, the average of three readings taken at various locations was used to evaluate the contact angle.

### 2.4. In Vitro Study

#### 2.4.1. Cell Culture

In this experiment, mouse L929 fibroblast cells were cultured in a medium consisting of alpha-MEM, 10% FBS and 1% PS (100 U/mL). The initial passage number of cell was 5. After every 48 h, the media were replaced and the cells were kept in an incubator at 37 °C with 5% humidity and 5% CO_2_.

#### 2.4.2. Scaffold Sterilization and Preparation of the Media

All the sponges were taken in 24-well plate cell culture dishes and sterilized under UV radiation in the clean bench for 2 h. Thirty mg of each sample were placed into a 15 mL tube containing 10 mL of alpha-MEM medium with an incubation period of 24 h at 37 °C then centrifuged at 5000 rpm for 5 min. Finally, the extracted media in different tubes was separated from sample sediment at the bottom.

#### 2.4.3. In Vitro Biocompatibility Study

The standard protocol [ISO16886] was followed for the MTT colorimetric assay to check the cytotoxicity of the cells in each fabricated sample [37]. The cells were seeded in 24-well cell culture plates with 1 mL of a L929 fibroblast suspension (approximately 2 × 10^4^). The culture medium was changed after every 48 h. A hemocytometer was used to count the cells. After 1, 3 and 7 days, the cell medium was aspirated and an MTT solution (5 mg/mL) in a 1:10 ratio was added to all compositions. After 4 h of incubation the MTT was removed. Then, 400 μL of DMSO was added to each well plate and incubated for 30 min for the dissolution of formazan crystals. All samples then transferred to a 96-well plate. Lastly, absorbance was measured by ana ELISA reader (Infinite F50, TECAN, Männedorf, Switzerland) at a 570 nm wavelength. Three samples of each section were examined to interpret the cytotoxicity. Cell viability (%) from the MTT graph was calculated using Equation (2):

(2) $$\mathrm{Cell}\;\mathrm{viability}\;\left(\%\right)\;\mathrm{after}\;7\;\mathrm{days}=\frac{\mathrm{Absorbance of samples after 7 Days}}{\mathrm{Absorbance}\;\mathrm{of}\;\mathrm{control}\;\mathrm{after}\;7\;\mathrm{days}}\times 100$$

At the start of the cell proliferation study, a cell suspension (approximately 2 × 10^4^) was seeded in 24-well sized cell culture dishes. All sample extracts were used in each petri dish for all sets of samples. Then the dishes were kept at 37 °C in a humidified incubator with 5% CO_2_ for 1, 3 and 7 days. After the incubation period, a one-time rinsing and fixing with PBS was carried out with 4% PFA for 10 min. Later, three times PBS washes were done for permeabilization of fixed cells with 0.5% Triton-X100 for 10 min and non-specific blocking with 2.5% BSA for 1 h. FITC (25 μg/mL) was applied for 3 h, which bound and labeled the actin cytoskeleton of the attached cells. To stain the nuclei, HOECHST-33342 (1 μg/mL) was applied for 10 min. After complete washing and air drying, one drop of mounting solution was added to each dish. The samples were then analyzed under a fluorescence microscope (Olympus, FV10i-W, Tokyo, Japan) and images were captured.

#### 2.4.4. Live and Dead Assay

A live and dead assay was performed using a LIVE/DEAD™ Viability/Cytotoxicity Kit (Thermo Fisher scientific, USA) following the manufacturer’s protocol. At first, nearly 2 × 10^4^ L929 cells were seeded and cultured for 1, 3 and 7 days. The staining solution contained 10 mL of PBS, 20 μL of ethidium homomodimer-1 (EthD1), and 5 μL of calcein AM. Each sample received 200 µL of the test solution, which was then incubated for 45 min at 20–25 °C. A fluorescence microscope (Olympus, FV10i-W, Tokyo, Japan) was used for visualization. Live and dead cells were calculated following formula (3),

(3) $$\mathrm{Cell}\;\mathrm{viability}\;\left(\%\right)=\frac{\mathrm{Total}\;\mathrm{cells}-\mathrm{Dead}\;\mathrm{cells}}{\mathrm{Total}\;\mathrm{cells}}\times 100$$

#### 2.4.5. In Vitro Hemolysis Assay

A hemolysis experiment was performed to get an idea of how biocompatible the scaffold was in the in vitro study. For this assay, 10 mL fresh blood was taken in a tube from an SD rat containing an anticoagulant acid citrate dextrose (ACD) solution at a ratio of 9:1 and centrifuged it at 3000 rpm for 10 min at 25 °C to separate the blood components. Isolated cells from blood plasma were rinsed two times in PBS and centrifuged again to obtain the RBC sediment. From the blood sediment, 2% erythrocyte solution (0.2 mL) was prepared in 10 mL PBS extract for every sample. Thereafter, the mixture was centrifuged again as mentioned. Triton-x100 (10 mL 0.1% Triton-x100 + 0.2 mL 2% erythrocyte susp), and PBS (10 mL PBS + 0.2 mL 2% erythrocyte susp) were marked as the positive and negative controls, respectively. In the end, absorbance samples were recorded by a microplate reader (Infinite F50, Tecan, Männedorf, Switzerland) at 540 nm. The hemolysis ratio (HR%) can be calculated using Equation (4):

(4) $$\mathrm{HR}\%=\frac{\mathrm{AS}-\mathrm{AN}}{\mathrm{AP}-\mathrm{AN}}\times 100$$

where, AS = absorbance of sample supernatant; AN = absorbance of negative control; and AP = absorbance of positive control [38].

#### 2.4.6. Evaluation of Coagulation Capacity

Red-blood-cell attachment and platelet adhesion were studied by treating different specimens in whole blood. At first, all sponges were cut into 1 × 1 cm squares and placed in a 12-well plate. To evaluate RBC attachment, whole blood droplets were directly added to the scaffolds, and then the plate was incubated at 37 °C for 20 min. To remove physically adhered cells, it was washed three times with PBS. The PBS-washed sponges were fixed with 2.5% glutaraldehyde solution for 2.5 h, and ethanol was gradually introduced for dehydration at different concentrations: 50, 60, 70, 80, 90 and 100%. Each concentration was changed after 10 min. Composites were air dried at room temperature. The final dried samples were placed in an SEM sample holder to visualize the microstructure.

To get platelet-rich plasma (PRP), whole blood was centrifuged at 2000 rpm for 10 min. Then PRP was introduced immediately into 1 × 1 cm samples in 12-well plates and incubated for 60 min at 37 °C. The aforementioned procedure was then carried out, and scaffold interaction was visualized through SEM images.

### 2.5. In Vivo Study (Rat Tail Amputation Model)

To perform the in vivo rat tail model experiment, we used 12-week-old rats. Initially, they were all anesthetized by Isofurane (Terrell, TX, USA) with adequate oxygen supply. The tails were properly sterilized with 70% ethanol. Fabricated hemostatic samples were cut into 5 × 5 mm squares using a biopsy punch. An injury was generated in the tail with the support of a scalpel and convenient forceps while taking all necessary measures. A stopwatch was used to record total bleeding time for all the applied scaffolds. Pre-weighted surgical gauge was applied to the wound to absorb blood until the bleeding stopped.

### 2.6. Statistical Analysis

The mean and standard deviation of the results were shown as (±SD). For the comparisons, statistical significance was examined via one-way and two-way ANOVA tests and then the Tukey post-hoc test using Graph pad Prism software (version 8.0). The results were significant at *p* < 0.001 ***, *p* < 0.01 **, and *p* < 0.05 *.

## 3. Result and Discussion

### 3.1. Morphology and Microarchitecture Analysis

**Figure 1:**
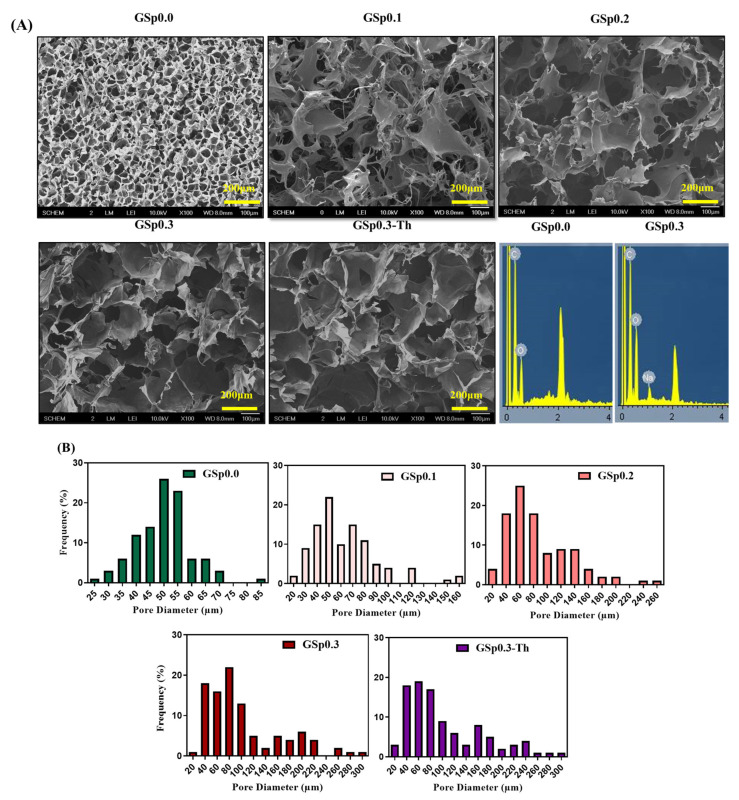
(**A**) SEM cross section micrographs of novel (GSp) scaffolds, EDS of GSp0.0 and GSp0.3, (**B**) Pore-size diameter distribution of GSp0.0, GSp0.1, GSp0.2, GSp0.3, GSp0.3-Th.

Figure 1A represents cross-sections of the lyophilized sponge samples (GSp0.0, GSp0.1, GSp0.2, GSp0.3, GSp0.3-Th) by SEM. The pores of each sample were unique. Gsp0.0 had a rigid shape and identical pore size. After increasing Sp concentration, GSp0.1, GSp0.2, GSp0.3, GSp0.3-Th pores gradually become more uniform, porous, stable and well inter-connected on account of the interaction of crosslinked Sp with G. The SEM images of the samples’ microstructures contained both micropores and macropores. This fine porous structure was ultimately essential for cell attachment, gaseous molecule exchange and systemic circulation supply [38]. 

EDS verified that the sponge contained the necessary components. Desirable elements like Carbon (C) and Oxygen (O) were detected in GSp0.0. Furthermore, the GSp0.3 group showed sodium (Na) alongside C and O.

Figure 1B reveals pore diameter vs frequency percentage. In GSp0.0, GSp0.1, GSp0.2, GSp0.3, GSp0.3-Th, sponges had pore sizes of 25–85, 20–160, 20–260, 20–300 and 20–300 μm, respectively. Typically, each scaffold had a pore diameter ≤300 μm. Due to SAP characteristics and an increased concentration of Sp, the pores become larger. Small pores of hemostatic materials facilitate diffusion, clotting and molecular transport, whereas large porous structures are highly effective at absorbing blood from bleeding sites; thus, they serve a hemostatic function.

### 3.2. Fourier Transform Infrared Spectroscopy (FTIR) 

**Figure 2:**
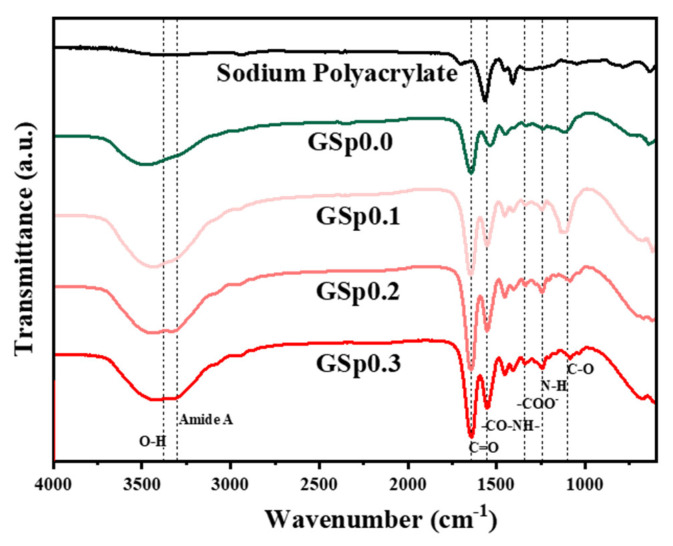
Functional groups identification through FTIR.

Figure 2 displays representative FTIR spectra of raw materials (Sp, G) and composites (GSp0.1, GSp0.2, GSp0.3). Basically, the graph reveals the functional groups of each sample. At 1100 cm^−1^, a distinctive C–O bond stretched gradually from GSp0.0 to GSp0.3. A typical symmetric bending of amide II (–CO–NH–) occurred at 1556 cm^−1^ and the vibration of –COO⁻ at 1339 cm^−1^ confirmed Sp presence [39]. A prominent peak of amide I (C=O) at 1643 cm^−1^, amide III (N–H) at 1240 cm^−1^ and an amide A group at 3300 cm^−1^ represented gelatin. The O–H spectrum also appeared at 3380 cm^−1^ and showed G confirmation. In GSp, the sponge peak intensities of amide II (1556 cm^−1^) and amide I (1643 cm^−1^) merged, became stable and followed an ascending trend due to the mixing of raw materials during fabrication.

### 3.3. PBS Uptake Behavior

**Figure 3:**
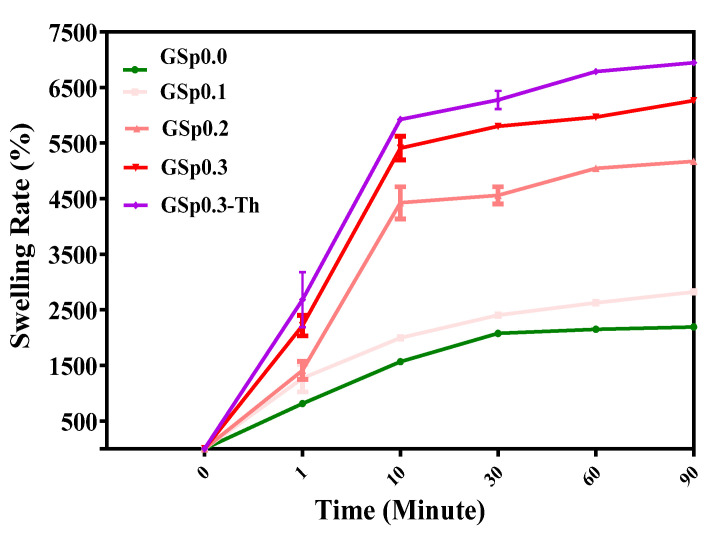
Graphical representation of swelling rate (%).

A superabsorbent polymer is a kind of hydrogel that can swell up to thousand times its own weight, whereas the absorption capacity of common hydrogels is not significant compared to SAP. In Figure 3, the swelling ratios of the samples followed an ascending trend that showed Gsp0.3-Th (6948%) had the highest swelling rate compared to Gsp0.0, GSp0.1, GSp0.2 and GSp0.3 (2194, 2822, 5171 and 6265%, respectively) due to the increasing amount of the polymer. The GSp sponge quickly absorbed water and attained its maximum swelling capacity in about 90 min. PBS uptake behavior was observed until the samples reached equilibrium and the scaffolds no longer gained any noteworthy weight. 

Meanwhile, it was necessary to understand why the SAP expanded so much. When hydrogen atoms are joined to small electronegative atoms like N, the O electrostatic interactions between molecules occurred and hydrogen bonds formed. The hydrogen atoms were joined to the lone pairs of non-bonding electrons on other nearby electronegative atoms. In water, the electronegative oxygen atom attracts the electrons of the hydrogen atom, creating a dipole in the molecule. On other water molecules, the lone oxygen pairs are joined to positive hydrogen atoms. Each of the two lone pairs of electrons in the oxygen is capable of forming a hydrogen bond with two other water molecules. These outcomes result in a decrease in system energy and an increase in entropy. Since an SAP is hydrophilic, its polymer chains have a tendency to spread in a given volume of water, increasing the number of configurations for the system and entropy [40]. An Sp is a long-chained copolymer generated from the interaction between AA and SA [41]. This acrylic crosslinked synthesized chemical has sodium in its core structure [42] and has repeating polyelectrolyte anion and cationic carboxyl groups in its chain that have considerable water retention and can create a thick transparent gel. Once the superabsorbent has swelled with absorbed the water, the three-dimensional polymer network is wrapped in a membrane structure to hold the water. Hydrophilic structures have the highest ability to hold water. As a result, these porous scaffolds can readily soak up all blood from a bleeding zone. Therefore, they exchange gaseous molecules and other biological fluids between cells. Thus, it works as a potential hemostatic agent. 

### 3.4. pH Change Evaluation

**Figure 4:**
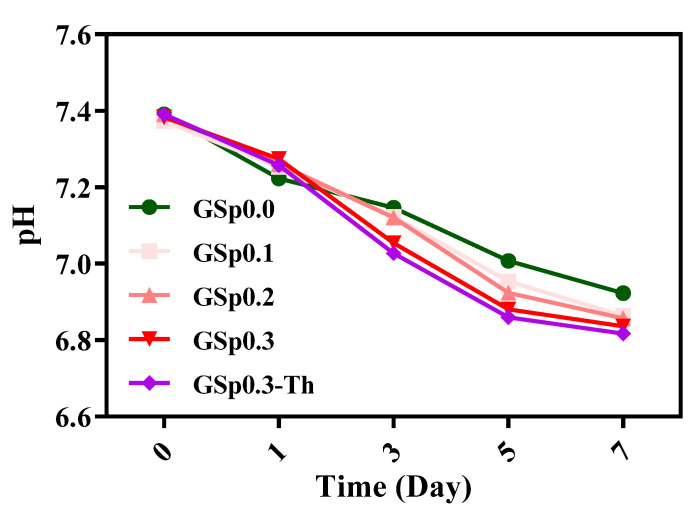
pH sensitivity evaluation vs. time graph.

pH change, monitored over seven days, had an insignificant effect on the capacity of cells to multiply and settle in the proper places inside the composites as shown in Figure 4. 

Initially, all the pH samples were 7.4 but with time they gradually fell: GSp0.0, Gsp0.1, Gsp0.2, GSp0.3, GSp0.3-Th (6.92, 6.86, 6.85, 6.83 and 6.81 respectively). Thus, pH shifted from neutral (7.4) to neutral-medium GSp0.3-Th (6.8) after 7 days following a descending trend due to repeated amounts of –COO– groups, which lessen interactions between hydrogen atoms. The difference was deemed negligible compared to the control GSP0.0, which showed that the materials released few hydrogen ions. This demonstrated that the following sponges are capable of withstanding pH changes.

### 3.5. Water Contact Angle

**Figure 5:**
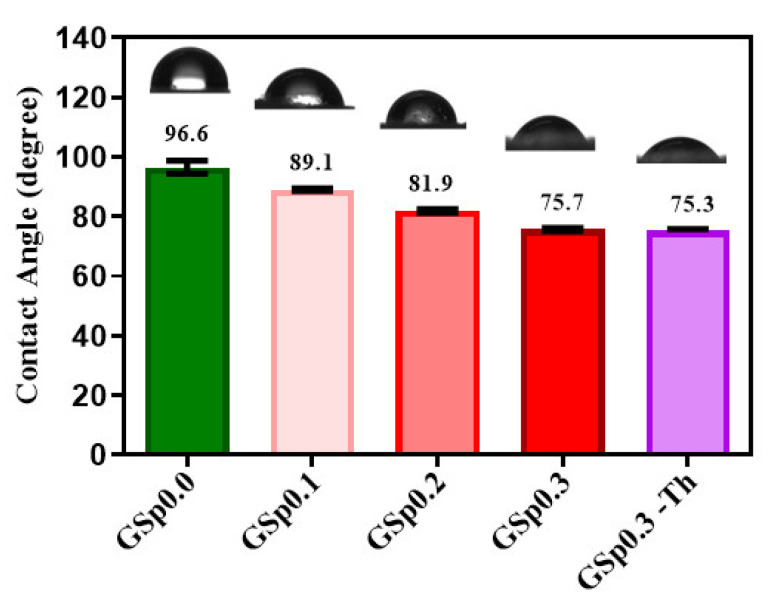
Hydrophilicity observation by water contact angle.

Surface wettability was evaluated by the water contact angle, which is essential for cells to adhere to biomaterials followed by cell migration, proliferation, and viability. Serine, threonine, asparagine, glutamine, aspartic acid, and glutamic acid are examples of hydrophilic amino acids that remain in G while hydrophobic amino acids, such as leucine, valine, phenylalanine, isoleucine, and methionine, are preferentially realigned due to their hydrophobic nature, resulting in the formation of a hydrophobic surface on GSp0.0 (96.6 ± 4.210) [43]. Numerous hydrophilic groups, including hydroxyl, amine, and carboxyl moieties, can be found in Sp. Introducing naturally occurring or hydrophilic polymers into synthetic polymers might be an intriguing strategy to enhance hydrophilicity [44]. However, all of the manufactured grafts present water affinity by measuring the contact angles of the samples as seen in Figure 5. 

The water contact angles for the composites were GSp0.1 (89.10 ± 0.9695), GSp0.2 (81.93 ± 1.305), GSp0.3 (75.73 ± 1.097), GSp0.3-Th (75.33 ± 0.8342), and these followed a descending trend that clearly expressed the increasing hydrophilicity of sponges. When the contact angles are more than 90° and less than 90°, the materials are referred to as hydrophobic and hydrophilic, respectively. The high surface tension of water is what causes substances to change from hydrophilic to hydrophobic [45,46]. As a result, a sample’s porous structure and effectiveness as a hemostatic agent are closely correlated with its hydrophilicity.

### 3.6. In Vitro Biocompatibility Study

**Figure 6:**
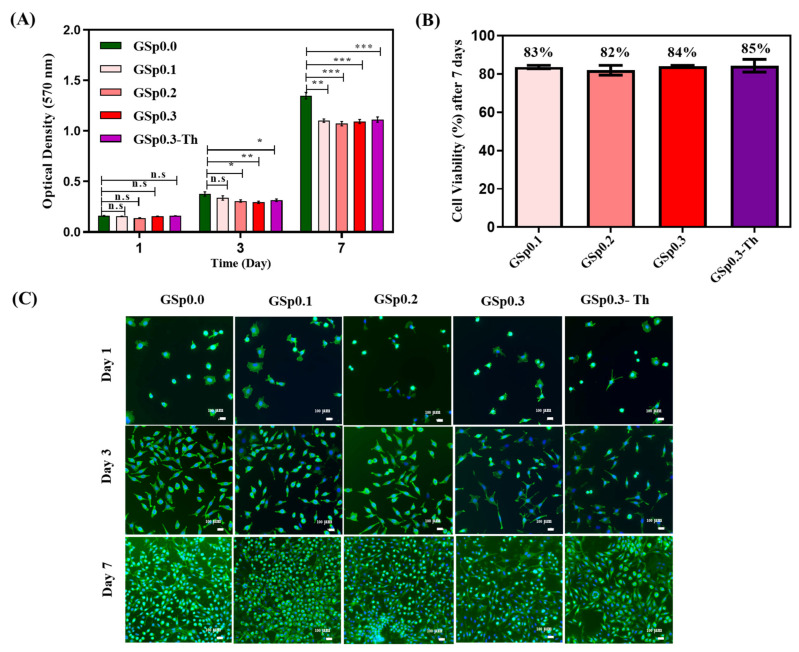
(**A**) MTT test over span of 7 days, (**B**) Cell viability after 7 days calculated from MTT assay, (**C**) Fluorescence images of attached fibroblast L929 cells (scale bar 100 µm; n.s.: not significant; *p* < 0.001: ***; *p* < 0.01: **; *p* < 0.05: *).

A quantitative evaluation of cytocompatibility was performed using the MTT Assay. Figure 6A illustrates the assay in fibroblast cells. The cell-proliferation rate was evaluated using the MTT test. Here, optical density (OD) was plotted against time (days) in the graph and absorbance was taken at 570 nm after 1, 3 and 7 days for each scaffold. GSp0.0 was considered as control. Although there was no noticeable difference after 1 day of incubation, cell growth accelerated considerably after 3 and 7 days due to elevated metabolic activity. A week later, GSp0.2, GSp0.3, GSp0.3-Th showed the highest proliferation rate (*p* < 0.001: ***). Compared with control at the same time, the GSp0.1 cell growth rate was (*p* < 0.01: **).

Figure 6B represents the cell viability percentage after 7 days calculated from the MTT graph. Here, every sponge showed the (%) viability > 80% when compared to control GSp0.0, demonstrating that all samples are biocompatible.

For observing the morphology and fibroblast fluorescence cell-attachment images Figure 6C were taken. Green and blue colors identify the cell F-actin and nuclei, respectively. The findings demonstrated that the nature of G has a significant effect on the culture and proliferation of fibroblasts in wounds. After one week, mitosis was shown by the scattered spindle shaped filamentous L929 cells, which showed that the cells were healthy. Thus, GSp scaffolds had sustainable capacity for cell proliferation. 

### 3.7. Live and Dead Assay

**Figure 7:**
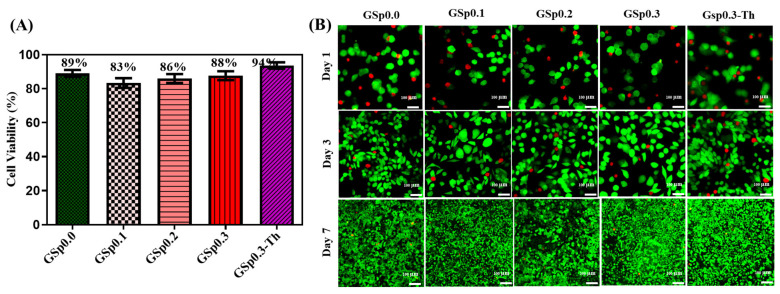
(**A**) Total L929 cell viability (%) of GSp samples calculated from live and dead images, (**B**) Live and dead cells fluorescence images of different scaffolds; scale bar 100 µm.

In a polymer drug-delivery system, biocompatibility plays a central role. Since the percentage of cell viability shown in Figure 7A is more than 80% in the GSp0.0, GSp0.1, GSp0.2, GSp0.3 and GSp0.3-Th hemostat composites, the samples are marked as nontoxic [47]. In this study, viability was calculated from live and dead cells images using (National Institutes of Health, NIH) Fiji Software. Figure 7B represents the fluorescence images of fibroblast cells. The live and dead cells are identified by green and red dots, respectively. 

### 3.8. In Vitro Hemocompatiblity Study

**Figure 8:**
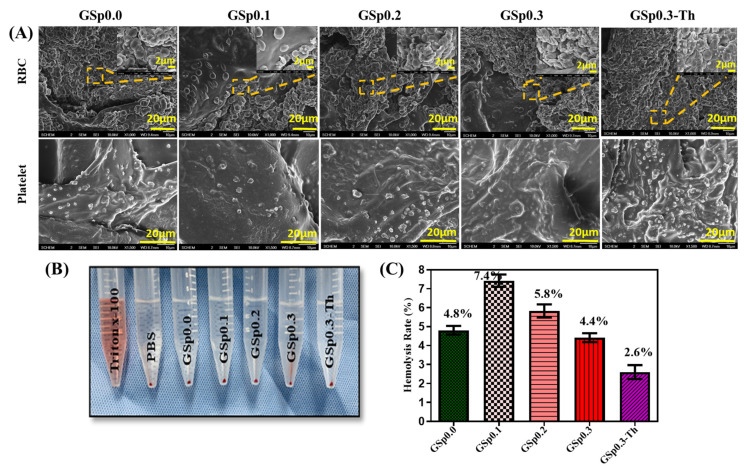
(**A**) SEM images of erythrocyte aggregation (low magnification 1000×, high magnification 4000×), platelet adhesion (magnification 1500×), (**B**) Photograph of hemolytic activity, (**C**) Hemolysis percentage of GSp scaffolds.

For further assurance, in vitro blood clotting activity was tested. Here, Triton x-100 and PBS was taken as positive and negative controls, respectively. The HR% indicated the degree to which red blood cells broke down when in close proximity. A hemolysis ratio of less than 5% was regarded as blood-compatible by ISO 10993-4 [47]. HR% is shown in Figure 8C where GSp0.0 (4.804%) and GSp0.3 (4.417%) exhibited activity within the limit. The addition of thrombin to the composite GSp0.3-Th showed the lowest HR% (2.601%) due to the synergistic interaction of thrombin, Sp and gelatin. Thrombin is an active hemostatic agent, so it has the ability to activate and speed up the coagulation cascade directly, thus improving hemostatic potency in an active pathway. Passive hemostatic materials had good biocompatibility and sped up hemostasis without affecting the coagulation cascade. The hemostatic potential of the scaffold was thought to be significantly increased by the addition of active hemostatic Th to passive hemostatic materials. However, the widespread use of these active hemostatic agents in a variety of situations has been restricted by possible virus infection, thrombosis, or general or systemic emboli. To eliminate these dangers, recombinant Th was created, which can be employed alone, in conjunction with fibrin, or in conjunction with another hemostatic agent to achieve hemostasis [31,48]. 

SEM images were used to visualize erythrocyte and platelet aggregation as shown in Figure 8A. Hemostasis was significantly influenced by platelets and erythrocyte attachment. RBCs having a polyhedron shape may have resulted in seamless aggregate sealing. Thus, it was essential to pay attention to the red blood cell and platelet attachment on the scaffolds in whole blood. The aggregation of platelets on the sample surface-initiated activation. With an increasing concentration of Sp and added Th, more RBCs became attached to the scaffold surface. Th-loaded sample GSp0.3-Th exhibited considerable erythrocyte accumulation and a higher proportion of platelets adsorbed on the surface comparing to GSp0.0. Sometimes the interaction of newly prepared biomaterials with blood led to unwanted inflammation and fibrinolysis. Therefore, creating scaffolds with higher hemocompatibility raises tolerability and reduces undesirable side effects, such as thrombus formation [48]. Hence, it can be said that GSp scaffolds produced a quick hemostatic impact with better erythrocyte and platelet adsorption and were not harmful to blood cells.

### 3.9. In Vivo Rat Tail Model

**Figure 9:**
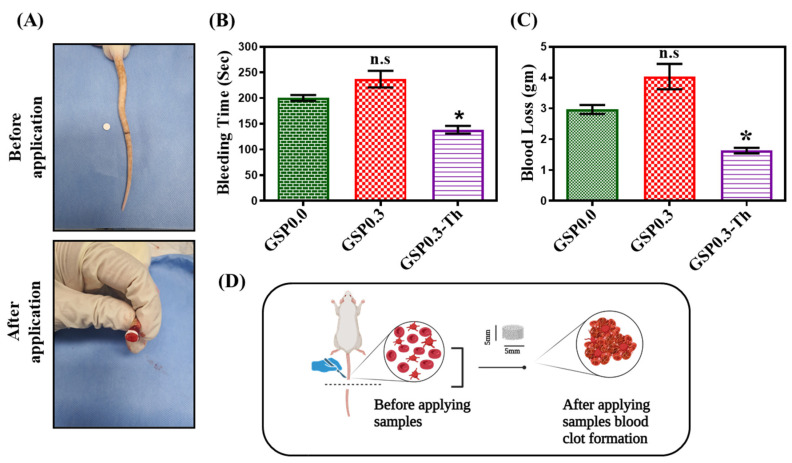
(**A**) Use of GSp scaffolds in rat tail amputation model, (**B**) Hemostatic time, (**C**) Blood loss from wound site (n.s.: not significant, *p* < 0.05: *), (**D**) In vivo before and after application of samples and hemostatic mechanism.

Hemostatic time and blood loss were calculated through the rat tail amputation model. In Figure 9B blood-clotting times were (200 ± 10 sec) and (237 ± 28 sec) respectively, for GSp0.0 and GSp0.3. More impressively, the addition of Th in GSp0.3-Th fell to (138 ± 13 sec). A previously weighted gauge was used to assess how much blood was lost. Samples of blood loss activity are shown in Figure 9C in GSp0.0 (2.967 ± 0.2517 gm), GSp0.3 (4.033 ± 0.7095 gm) and GSp0.3-Th (1.633 ± 0.1528 gm). GSp0.3-Th showed the lowest blood absorption. Hemostatic chemicals played a crucial role in ensuring quick blood clotting in the preliminary stages of damage. The main mechanism was initial hemostatic stage activating secondary blood coagulation cascades where fibrinogen transformed into insoluble fibrin. Then platelets and fibrin threads combined to create a blood clot [49]. The addition of biodegradable gelatin to synthetic sodium polyacrylate polymer improved the clotting properties, while the mixing of Th facilitated more rapid blood-clotting during dressing application. 

## 4. Conclusions

In summary, quick blood clotting is essential for a safe and effective hemostatic agent. GSp scaffolds displayed bigger and closely connected pores and a high water retention capacity because of the synthetic superabsorbent biomaterial and because G has a profound impact on how fibroblasts form and multiply. Based on these findings, a considerable rate of fibroblast L929 cell proliferation was demonstrated by in vitro cytocompatibility. The research of in vitro whole blood coagulation capacity demonstrated that GSp0.3 generated larger blood clots that dramatically triggered the trapping of more RBCs after loading Th in GSp0.3. Therefore, GSp0.3 and GSp0.3-Th exhibited maximum activity in almost every approach compared to the Gsp0.0, GSp0.1, GSp0.2 composites. Thus, the scaffold GSp0.3-Th was more effective at controlling bleeding both in vitro and in vivo. Overall, our research revealed a promising functional hemostatic agent composed of both natural and synthetic biomaterials that is readily accessible and reproducible.

## Figures and Tables

**Table 1:** Representation of various composite groups.

Name	Gelatin, G (g/L)	Sodium Polyacrylate, Sp (g/L)	Thrombin, Th (NIH/mL)
GSp0.0	20	-	-
GSp0.1	20	1	-
GSp0.2	20	2	-
GSp0.3	20	3	-
GSp0.3-Th	20	3	50

## Data Availability

Not applicable.
